# Supplementary material for: Small-Extracellular-Vesicle-Derived miRNA Profile Identifies miR-483-3p and miR-326 as Regulators in the Pathogenesis of Antiphospholipid Syndrome (APS)
Source: Int J Mol Sci. 2023 Jul 18;24(14):11607. doi: 10.3390/ijms241411607 (PMC10380201; doi:10.3390/ijms241411607)
Supplement: Supplementary file 1 [file ijms-24-11607-s001.zip › ijms-2449981-supplementary.pdf]

Supporting Information

Small extracellular vesicles derived miRNA profile identifies miR-483-3p and miR-326 as regulators in the pathogenesis of primary antiphospholipid syndrome (PAPS)

Cristina Solé [1*], Maria Royo[1], Sebastian Sandoval[1], Teresa Moliné[2], Josefina Cortés-Hernández[1]

[1] Rheumatology Research Group - Lupus Unit, Vall d'Hebrón University Hospital, Vall d'Hebrón Research Institute (VHIR), Universitat Autònoma de Barcelona (UAB), Barcelona, Spain. [2] Department of Pathology, Vall d'Hebrón University Hospital, Barcelona, Spain.

INDEX

1. SI Materials and Methods 3

2. References 10

3. Supplementary Figures 11

4. Supplementary Tables 22

1. SI Materials and Methods

Patients' clinical characteristics

A total of 50 primary PAPS patients fulfilling the International Consensus statement criteria [1], 30 persistent aPLs without associated thrombotic or obstetric complications and 30 healthy donors were included in the study after ethics committee approval was obtained. All patients provided written informed consent.

We excluded APS patients who had evidence of an underlying systemic disease or antibodies against double-stranded DNA or extractable nuclear antigen. PAPs included those with objectively confirmed arterial or venous thrombosis and a positive result on aPL testing on 2 occasions at least 3 months apart. Testing for aPLs was performed at Vall Hebron Hospital. Included patients had lupus anticoagulant or moderate to high titres (≥40 GPL [IgG phospholipid] units) of IgG anticardiolipin, anti–β2-glycoprotein I antibodies, or both, measured in accordance with international guidelines [1-3]. None of the controls had a history of autoimmune disease, bleeding disorders, thrombosis, or pregnancy loss.

Antiphospholipid antibody measurement

Anticardiolipin antibodies and anti-β2-glycoprotein I antibodies (isotypes IgG and IgM) were quantified following the manufacturer's instruction of AESKULISA Cardiolipin-GM and AESKULISA β2 Glyco–GM (Aesku, Wendelsheim, Germany). They are solid phase enzyme immunoassay for the quantitative and qualitative detection of IgG and IgM antibodies against cardiolipin or β2 glycoprotein I in human serum, respectively. Because of the lack of WHO reference material, AESKULISA Cardiolipin-GM is calibrated against reference sera from N.E. Harris, Louisville. The results are expressed in GPL/ml for IgG and in MPL/ml for IgM. For AESKULISA ß2-Glyco-GM, the assay is calibrated in arbitrary units (U/ml). Both ELISA tests were standardised using the Sapporo-Standards HCAL for IgG and EY2C9 for IgM determination. Positive cut-off values were determined according to Sydney Criteria: threshold for positive anticardiolipin antibodies >40 GPL/MPL and for positive anti-β2 glycoprotein I antibodies >99th percentile of controls [3].

Lupus anticoagulant detection was determined by dilute Russell's viper venom time (DRVVT) and dilute APTT (DAPTT) in the plasma patient / plasma control mixture (1:1) in accordance with the international consensus guidelines [32]. The plasma control was a pooled plasma (PP)

home made by double centrifugation to ensure that the PP contains minimal residual platelets and around 100% activity for all clotting factors. Positive values were considered the value above the 99th percentile of controls. Positive results at diagnosis were confirmed after 12 weeks to exclude transient LA induced by infection or drugs following Sydney criteria [3].

Blood Samples collection

Blood samples were also extracted for serum, plasma collection and PBMCs isolation at diagnosis. Blood samples were collected in SST serum separation tubes (Vacutainer SST, BD Biosciences) for serum collection and centrifuged at 2,000 *g* during 20min at room temperature. Samples were collected in sodium citrate tube 3.2% (Vacutainer, BD Biosciences) and plasma was obtained after double centrifugation at 2,000 *g* for 15 min at 4°C and at 16,000 *g* for 10 min at 4ºC. Then samples were stored at−80°C until use.

For PBMCs isolation, blood samples were collected in mononuclear cell preparation tubes with sodium citrate (Vacutainer CPT, BD Bioscences). After 30 min of centrifugation at 3000 rpm the section containing PBMCs was clearly visible, and cells were collected using a pipette and washed twice in phosphate buffered saline (PBS). The cellular pellet was frozen at -80ºC with cell culture freezing medium for monocyte *in vitro* experiments.

sEVs Characterization by Cryo-TEM, NanoSight, Western-Blot and flow cytometry

The characterization by cryo-TEM and NanoSight was done at the Universitat Autonoma of Barcelona (UAB, Barcelona, Spain). For cryo-TEM characterization, a drop of a dilution of exosome pellet (1:100 in PBS) was put on a frozen grid. This was transferred to a cryopreparation chamber (Leica EM CPC, Barcelona, Spain) using a GATAN cryotransfer apparatus to prepare the sample for freezing with propane and ethane. The temperature of the grid was maintained at −174 °C. The vitrified samples were examined using a JEM-1400 electron microscope (Jeol, Peabody, MA, USA) with an acceleration voltage of 40–120 kV. For NanoSight characterization, different dilutions of exosomes were examined using the Nanosight LM-20 particle size analyser (Malvern Panalytical, Malvern, UK).

The protein isolation of exosomes was carried out using the Total Exosome RNA & Protein Isolation Kit (Applied Biosystems, Foster City, CA, USA). For Western blot analysis, the samples were loaded on 10% polyacrylamide gels and transferred to the membrane. Different antibodies against exosomal markers (rabbit anti-Alix and rabbit anti-TSG101, dilutions 1:1000,

Abcam, Cambridge, UK) were used to incubate the membrane and the proteins were detected using enhanced chemiluminescence detection reagents according to the manufacturer's instructions (Thermo Fischer, Waltham, MA, USA).

To determine the sEV cell origin by flow cytometry (LSR Fortessa, BD Biosciences, Erembodegem, Belgium), samples were labelled with CD41(αIIb), CD31 and CD16 fluorescent labelled antibodies to detect platelet, endothelial and monocyte-derived sEVs, respectively. CD62P antibody was used to evaluate platelet activation (Table S1). Incubation with conjugated monoclonal antibodies was of 20 minutes at room temperature.

sEVs quantification

A highly sensitive fluorescence-based enzymatic assay kit, FluoroCet Exosome Quantification Kit (System Biosciences, Palo Alto, CA) was used to quantify the number of small extracellular vesicles or exosomes of PAPS, aPL or HD samples according to manufacturer's instructions. Quantity of total exosomal protein was evaluated by Bradfor assay to obtain an aliquot between 600ng-1ug in a volume of 60uL. Lysis buffer (60ul) was added into sEV aliquots, and the mix was incubated at 4ºC during 30min to liberate sEV proteins. 50 µL standard or exosomal lysate, 50 µL working stock of buffer A, and 50 µL working stock of buffer B (making a 150 µL reaction volume) were mixed in each well of an opaque 96-well plate, incubated and protected from light for 20 min at room temperature. The fluorescence intensity (relative fluorescence units, RFU) was read using Fluorescence plate reader (Excitation: 530–570 nm and Emission: 590–600 nm). The blank for assay samples consisted of 1:2 PBS diluted FluoroCet lysis buffer, while the blank for standard reactions was 1:2 PBS-diluted FluoroCet reaction buffer. The experiment was repeated three times.

Procoagulant phospholipid (PPL) activity assay

The STA-Procoag-PPL (Stago, Asnieres, France) assay was used to evaluate the influence of isolated sEVs on the coagulation cascade activation. The PPL assay measures clotting time, in the presence of FXa and $CaCl_2$, of a system in which all factors are present in physiological levels (supplied by P-PPL Depleted Plasma) except the PPL which is supplied by the sample being tested. Isolated sEV pellet was diluted with 50µl of PL-Free plasma and triggered with 100µl of $CaCl_2$ and bovine Factor Xa solution. The clotting time was measured on an automated STA-Compact Max 2 Stago, Asnieres, France) following the manufacturer's protocol. Results are

expressed as coagulation time (sec), the shorter coagulation time the higher PPL activity. PPL assay was performed in batch with inclusion of positive and normal control samples, provided by the kit, for validation of the runs.

Isolation of primary monocytes and platelet from healthy donors

After centrifugation, PBMCs were extracted from healthy control blood samples (see sample collection). Monocytes were isolated from PBMCs using cold-aggregation method [4]. To separate monocytes from lymphocytes, PBMCs were resuspended in complete RPMI-1640 media (RPMI, 10% FBS, 10% Pen/Strep, 2 mM/L-Glutamine) and incubated for 30 min at 4ºC with continuous agitation. Monocytes rapidly aggregate in vitro at 4ºC obtaining a cellular pellet. The monocyte population was identified by flow cytometry based on forward and side scatter (FSC vs. SSC) gating, as well as on the expression or lack of the surface markers anti-hCD14-FITC (Dilution 1:100; Clone M5E2; BioLegend, USA; cat 982502) and anti-hCD3-Alexa 647 (Dilution 1:100; Clone OKT3; eBiosciences, USA; cat 51-0037-73).

For platelet isolation, 45mL of whole blood from healthy donors was obtained with a syringe containing 5 mL citrate buffer. Centrifugation was performed at 1500g for 15 minutes at room temperature and the upper phase was transfer to a 15 mL tube. To obtain the pellet platelet, the solution was centrifugated at 1500g for 5 minutes and after, the pellet was resuspended in 5-10mL Hepes to perform *in vitro* experiments. Fresh platelets were obtained and used within 2-3 hours.

sEV labelling and Internalization.

Isolated serum sEVs (100 µg) were fluorescently labelled using ExoGlow-Membrane EV Labelling kit (System Biosciences) according to manufacturer's instructions. Labelled sEVs were 10-fold diluted (10 µg), added into 24-well plates, and incubated with primary isolated cells for 2, 4, 6, 8 and 24 hours at 37°C. Then, cells were washed twice with PBS, fixed with 4% paraformaldehyde for 20 min and stained with 4′,6-diamidino-2-phenylindole (DAPI) for 5 min at room temperature. Finally, cells were washed and analysed under fluorescence microscopy (Olympus BX61). Uptake assays were performed in triplicate.

miRNA Extraction from sEVs

QIAzol (700 µL) was added to sEVs and immediately spun for 5 min at 5000×*g*; the lysate was collected and transferred completely into a new 2-mL tube. The lysate was incubated for 5 min

at room temperature (15–25 °C). Then, 90 µL of chloroform were added and incubated after vigorous shaking for 3 min at room temperature. The mixture was centrifuged at 12,000× *g* at 4 °C for 15 min. The upper aqueous phase was then transferred to a new collection tube, avoiding transferring any interphase. Two volumes of 100% ethanol were added and mixed. Then, 700 µL of the new sample were pipetted into a RNeasy MinElute spin column and centrifuged at 10,000× *g* for 15 s at room temperature. The step was repeated until completing volume transference. Once completed, the sample was spun for 15 s at 10,000× *g* after adding 700 µL of RWT buffer. One 15-s centrifugation cycle of 500 µL buffer RPE was then applied and another of 2 min was performed afterward. Then, the RNeasy MinElute spin column was centrifuged at full speed with an open lid to completely dry the membrane. Finally, 17 µL of RNase-free water were directly added to the centre of the spin column membrane; after letting it stand for 1 min, the sample was centrifuged for 1 min at full speed to elute the purified miRNA. Samples were then stored at −80 °C for later use.

miRNA-sequencing and analysis

miRNA extraction from aPL, PAPS or HD sEVs was performed following the instructions described previously (N=8). Quantification and evaluation of the miRNA quality was assessed by the Agilent 2100 bioanalyzer with Small RNA Chips. Small RNA-next generation sequencing was conducted at CNAG (Barcelona, Spain). RNA concentration was determined using a Qubit Fluorometer (Life Technologies, Paisley, UK). Libraries were performed using the NEBNext Multiplex sRNA Library Prep Set for Illumina kits (sets 1 [E7300S] and 2 [E7580S]; NEB, Ipswich, MA, USA). Reverse transcription primer was hybridised after 3’ adaptor ligation of 10 ng RNA per sample, following 5’ adaptor ligation. Twenty cycles of PCR were performed by Illumina feasible barcode primers after first strand cDNA synthesis. Subsequently, the barcoded samples were size selected on a 6% Novex Tris-Borate-EDTA (TBE) PAGE gel (Life Technologies, Paisley, UK), purified with the NucleoSpin Gel and PCR Clean-up kit (Macherey-Nagel, Düren, Germany), and quantified using the KAPA Library Quantification Kit (Kapa Biosystems, London, UK). Samples were pooled at equimolar ratio into two multiplex libraries containing 24 samples each. Next, the libraries were pre-amplified for four cycles and the appropriate size of purified fragments confirmed on the ScreenTape station. Clustering was performed on the Illumina cBot instrument, and samples were single end sequenced on HiSeq 2500 (HiSeq Control Software 2.2.58/RTA 1.18.64) with a 1 × 50 setup using the HiSeq Rapid SBS Kit v2 (all Illumina, San Diego, CA, USA). First step in miRNAseq analysis checked the quality of the sequencing experiments

using FASTQC tool. After that, alignment step was performed using Rsubread package [5] using *Homo sapiens GRCh38*, release 86, from Ensembl [6]. The DESeq2 (v1.24.0) Bioconductor's package [7] was used to determine differentially expressed ncRNAs (negative binomial distribution test, unpaired analysis). DESeq2 was used to calculate size factors and raw read counts scaled accordingly to normalize for library size. Lowly abundant sRNA species were filtered out if the sum of counts across all samples was less than 100. Reported p values were corrected for multiple comparisons using the Benjamini-Hochberg method. Adjusted p values < 0.05 were considered statistically significant. Differential expression was calculated between groups, although DESeq was used to calculate scale factors and normalised counts for all groups. Data is public on the Gene Expression Omnibus (NCBI) with access number GSE220791. The validated target genes for miRNAs were obtained from three major miRNA-target datasets: miRecords, miRTarBase, and miRWalk using the multiMiR R analysis Package.

miRNA cell transfection

Functional miRNA studies were performed in HUVECS and monocytes. Cells at ~70% confluence were transfected with mimic or anti-miR miRNA (mirVanaTM, Life technologies, Carlsbad, CA, USA) using Lipofectamine RNAiMAX Reagent (Life Technologies, Carlsbad, CA). We diluted lipofectamine with Opti-MEM Medium in one eppendorf and the mimic-483-3p or anti-miR-326 or their corresponding control (10µM) with Opti-MEM Medium in another eppendorf. Diluted mimic-miRNA or anti-miRNA and diluted lipofectamine were added to another eppendorf (1:1 ratio) for 5 minutes. After that, we added simiRNA-lipid complex to cells, and we incubated them for 24 hours.

miRNA gene expression

Taqman Advanced miRNA assay was used to quantify mature form of the miRNA from 1-10 ng of total RNA obtained from sEVs following manufacturer's instructions. To prepare the cDNA template, the kit Taqman Advanced miRNA cDNA Synthesis was used (Applied Biosystems, Foster City, CA). it consists of four reactions before performing the real-time PCR: 1) perform the poly(A) tailing reaction; 2) perform the adaptor ligation reaction; 3) perform the reverse transcription (RT) reaction and 4) perform the miR-Amp reaction. For perform the poly(A) tailing reaction RNA from each sample was mixed with 0.5 µl of 10x Poly(A) Buffer, 0.5 µl of ATP, 0.3 µl of Poly(A) Enzyme and 1.7 µl of RNase-free water. The reaction mixture was

incubated at 37°C for 45 minutes and 65°C for 10 minutes. After that, it was proceeded immediately to the adaptor ligation reaction. Reaction components were 3 µl of 5X DNA Ligase Buffer, 4.5 µl of 50% PEG 8000, 0.6 µl of 25X Ligation Adaptor, 1.5 µl of RNA Ligase and 0.4 µl of RNase-free water. The mixture was placed into a thermal cycler to incubate at 16°C for 60 minutes. Immediately, it was performed the reverse transcription (RT) adding 6 µl of 5X RT Buffer, 1.2 µl of dNTP Mix, 1.5 µl of 20X Universal RT Primer, 3 µl of 10X RT Enzyme Mix, 3.3 µl of RNase-free water. The reaction mixture was incubated at 42°C for 15 minutes and at 85°C for 5 minutes. As last step, the miR-Amp Reaction was performed using 25 µl of 2X miR-Amp Master Mix, 2.5 µl of 20X miR-Amp Primer Mix and 17.5 µl of RNase-free water. The reaction was incubated at 95°C for 5 minutes, followed by 14 cycles of 95°C for 3 seconds and 60°C for 30 seconds, and stopped at 99°C for 10 minutes second.

To perform the miRNA real-time PCR, we used the Taqman Advanced miRNA assay and Taqman Fast Advanced Master Mix (Applied Biosystems, Foster City, CA). The reaction was performed at 95°C for 20 second, followed by 40 cycles of 95°C for 1 second and 60°C for 20 seconds using QuantStudio 7 Pro PCR instrument (Applied Biosystems, Foster City, CA).

The data were normalised to miR-93-5p, which was the less variable miRNA between samples in the miRNA-seq analysis.

Western blot

HUVECs cells were incubated with sEVs from PAPS, aPL or HD (N=3) during 24 hours. After that, cells were lysed in 1x RIPA bufer (ThermoFisher Scientific, Carlsbad, CA, USA) containing Phosphatase Inhibitor Cocktail 2 and 3 (Millipore, Burlington, MA, USA) followed by western blot using Odyssey CLx Imaging System. Antibodies were typically duplexed using rabbit antibodies for phosphorylated antibodies and mouse antibodies for total protein. HRP secondary antibodies, Goat anti-Rabbit HRP and Goat anti-Mouse HRP, were used with the duplexed primary antibodies.

2. References

[1] Miyakis S, Lockshin MD, Atsumi T, et al. International consensus statement on an update of the classification criteria for definite antiphospholipid syndrome (APS). J Thromb Haemost 2006;4(2):295-306.

[2] Sciascia S, Sanna G, Murru V, et al. GAPSS: The Global Anti-Phospholipid Syndrome Score. Rheumatology (Oxford). 2013;52:1397-403.

[3] Pengo V, Tripodi A, Reber G, et al. Update of the guidelines for lupus anticoagulant detection. Subcommittee on Lupus Anticoagulant/ Antiphospholipid Antibody of the Scientific and Standardization Committee of the International Society on Thrombosis and Haemostasis. J Thromb Haemost 2009;7(10):1737-40.

[4] Chometon TQ, Siqueria MS, Santanna JC, et al. A protocol for rapid monocyte isolation and generation of singular human monocyte-derived dendritic cells. PLoS One 2020; 15(4):e0231132.

[5] Yang Liao, Gordon K. Smyth, and Wei Shi. The R package Rsubread is easier, faster, cheaper, and better for alignment and quantification of RNA sequencing reads. Nucleic Acids Research 2019;47(8):e47

[6] Frankish A, Vullo A, Zadissa A, et al. Ensembl 2018. Nucleic Acids Res. 2018;46(D1):D754-D761.

[7] Simon Anders and Wolfgang Huber. Differential expression analysis for sequence count data. Genome Biol. 2010;11(10):R106.

3. Supplementary Figures

Supplementary Figure S1. **Characterization and qualitative quantification of sEVs by western blot.** Representative western blots for the detection, as indicated, of the sEVs marker Alix and TSG101 in isolated sEVs from HD, aPL and PAPS patients. HD: healthy donors; aPL: patients with positive antiphospholipid antibodies without thrombotic or obstetric complications; PAPS: patients with primary antiphospholipid syndrome.

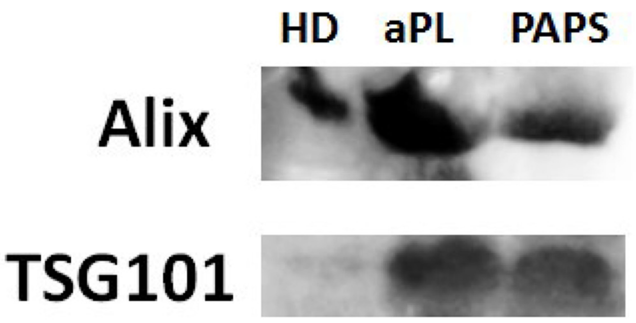

Supplementary Figure S2. **Quantification of sEVs in PAPS subgroup based on clinical manifestations or type of antiphospholipid antibody.** Abundance of isolated sEVs were measured by fluorescence emission at 590-600 nm with FluoroCet Exosome Quantification Kit. Quantity of sEVs did not differ in relation to clinical manifestations **(A)** or antiphospholipid antibody type or degree of positivity **(B)**. TMA: Thrombotic Microangiopathy; anti-B2GPI: anti-β2-glycoprotein I antibodies; anti-aCL: anti-cardiolipin; LA: lupus anticoagulant; triple positivity: positivity for LA, anti-aCL and anti-β2GPI. Comparison was performed by t-test student.

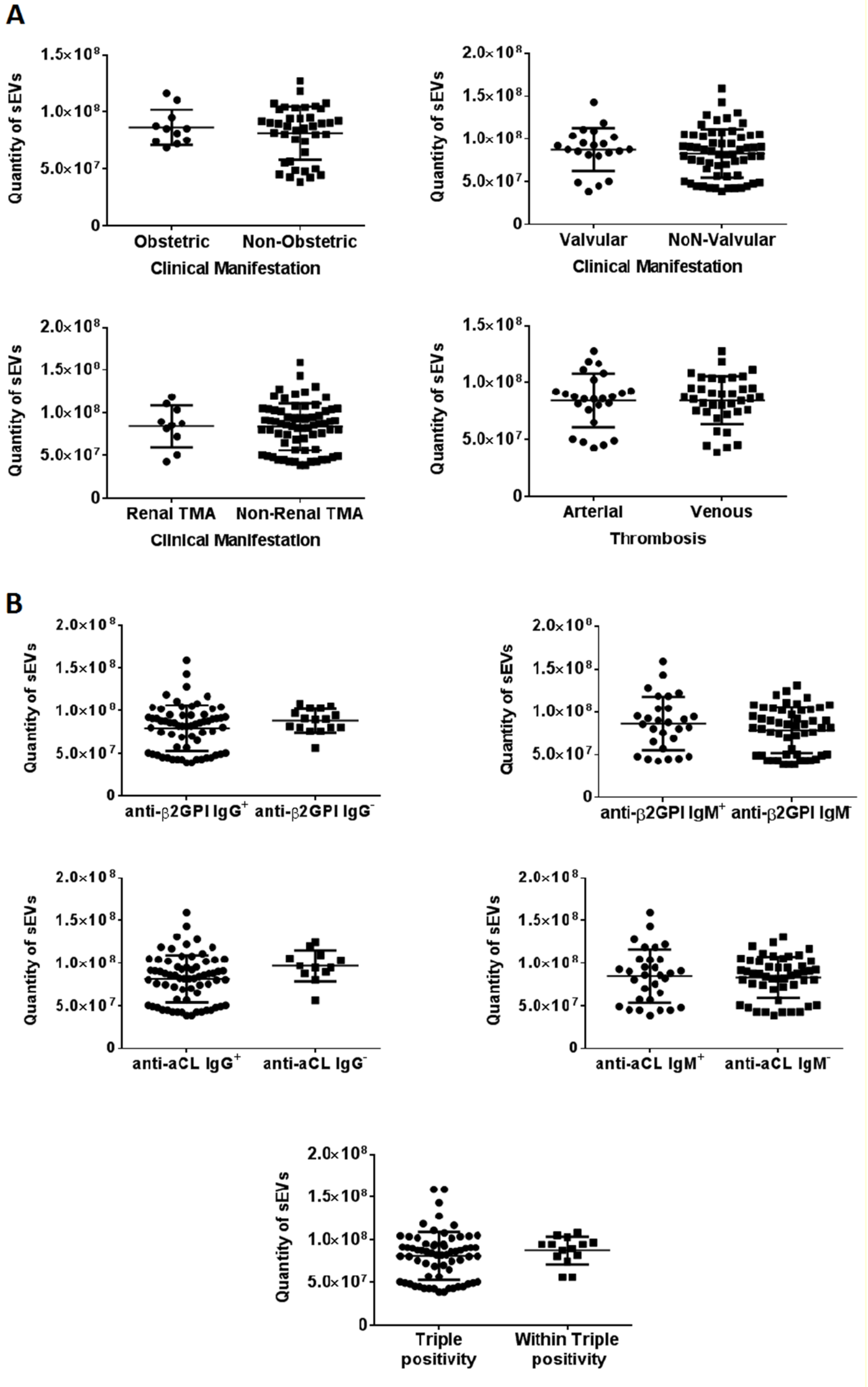

Supplementary Figure S3. Study of procoagulant potential of sEVs. Procoagulant phospholipid (PPL) activity was measured in plasma enriched with sEVs from PAPS, aPL or HD (N=10 for each group) by measuring PPL clotting time using a hemostatis analyser. PPL activity did not present difference betweensamples.

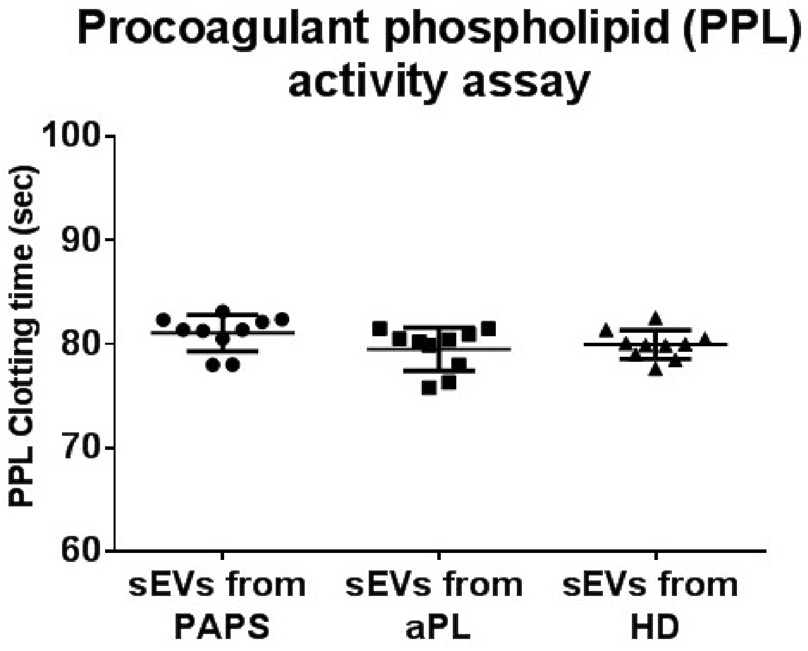

Supplementary Figure S4. Flow cytometry analysis for sEVs from PAPS, from aPL and HD. Surface markers of sEVs were analysed by flow cytometry (CD41+ for platelets, CD31 + for endothelial cells and CD16+ for monocytes). Representative forward scatter (FSC-A) vs side scatter (SSC-A) dot plot of the samples were performed to gate only small extracellular vesicles (size between 20-200 nm). Plasma derived sEVs are overall from platelet origin cell. No significant differences were observed between sEVs from PAPS, aPL or HD. sEVs: small extracellular vesicles; PAPS: with primary antiphospholipid syndrome; aPL: positive antiphospholipid antibodies; HD: healthy donors.

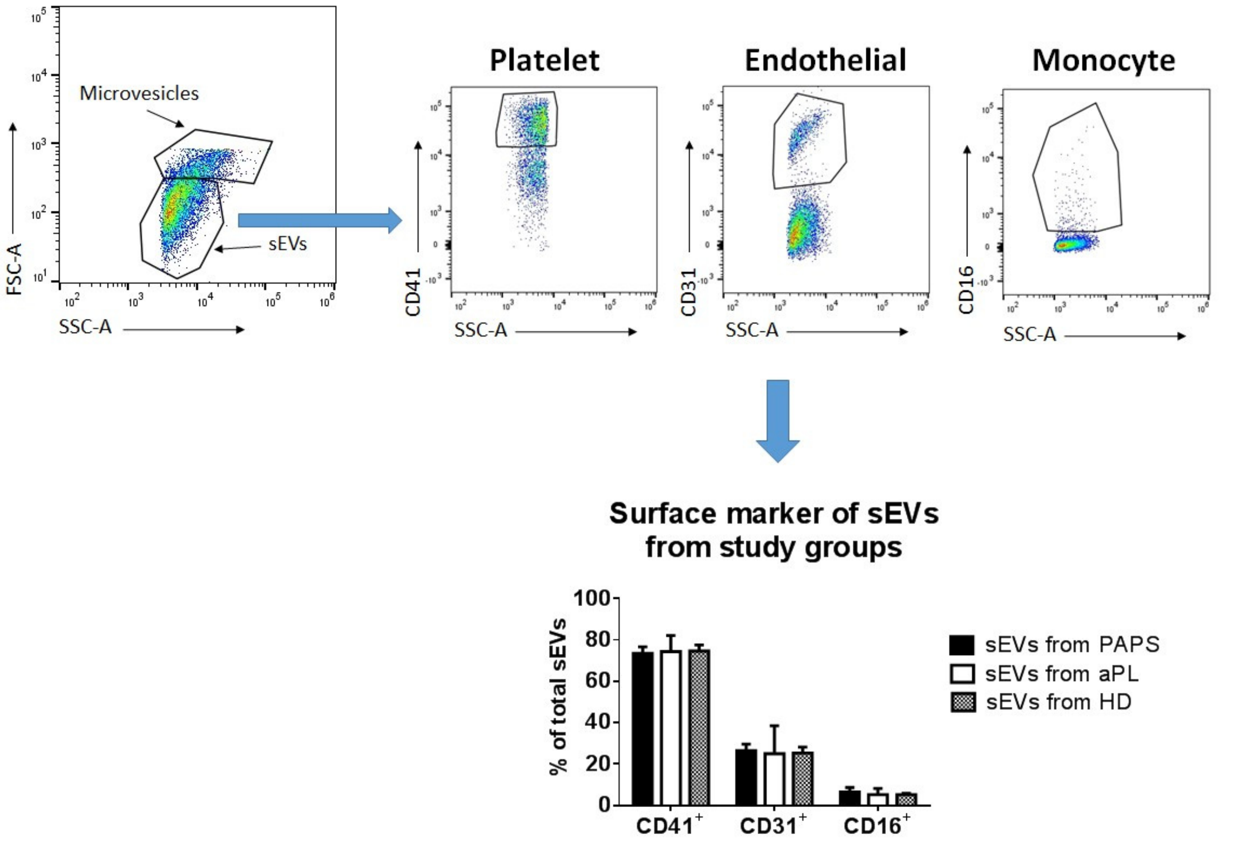

Supplementary Figure S5. ***In vitro*** **assessment of apoptosis in HUVECs after incubation with sEVs from PAPS, from aPL or HD.** Apoptosis was quantified by Annexin V-FITC/PI staining and evaluated by flow cytometry. The percentage of live cells, cells undergoing early, and late apoptosis were similar between groups. Experiments were performed in triplicate. sEVs: small extracellular vesicles; PAPS: with primary antiphospholipid syndrome; aPL: positive antiphospholipid antibodies and HD: healthy donors. Comparison was performed by one-way ANOVA test between samples.

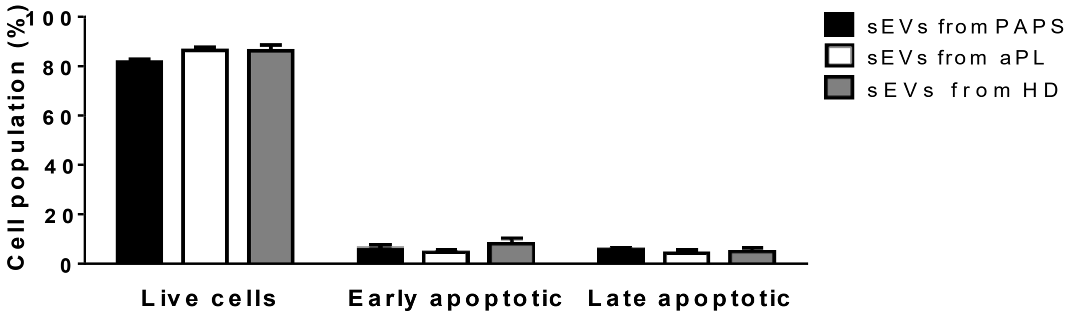

Supplementary Figure S6. Screening of proliferation and inflammatory pathways in HUVECs after incubation with sEVs from PAPS, from aPL or HD. qPCR-RT was performed to evaluate the gene expression of several biological pathways related in endothelial proliferation **(A)** or inflammation **(B)** after the incubation with isolated study sEVs. No significant differences were observed by one-way ANOVA test analysis. sEVs: small extracellular vesicles; PAPS: with primary antiphospholipid syndrome; aPL: positive antiphospholipid antibodies; HD: healthy donors and SELE: Selectin E gene.

A

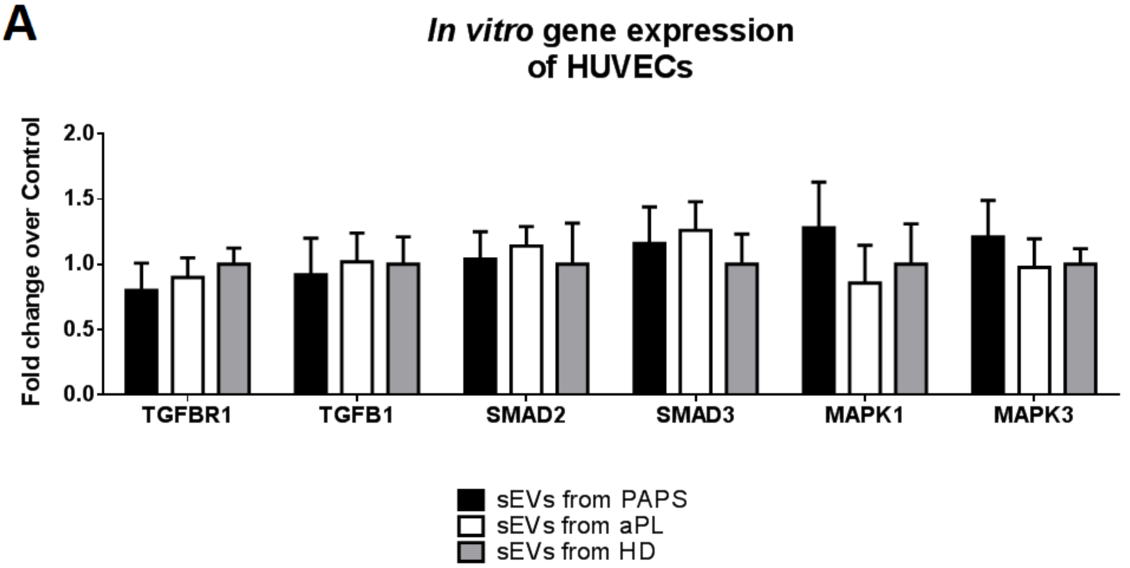

B

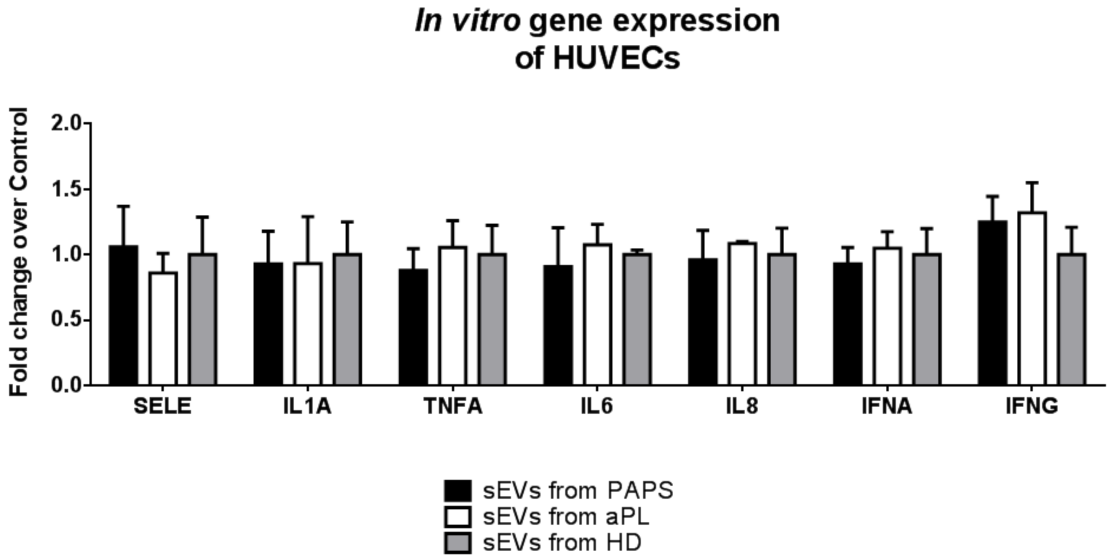

Supplementary Figure S7. Protein lysate of HUVECs after sEVs incubation from PAPS, aPL and HD. Wetern blot was performed to evaluate phosphorylation levels of MEK/ERK pathway. Tubulin- was used as control (Proteintech, mouse anti-human, 16801256) and p-ERK 1/2 (Thr202/Tyr204) antibody (Elabscience, rabbit anti-human, E-AB-70310) to evaluate phosphorylation protein. No significantly difference were observed. HUVECs: human Umbilical Vein Endothelial Cells; sEVs: small extracellular vesicles; PAPS: with primary antiphospholipid syndrome; aPL: positive antiphospholipid antibodies; HD: healthydonors.

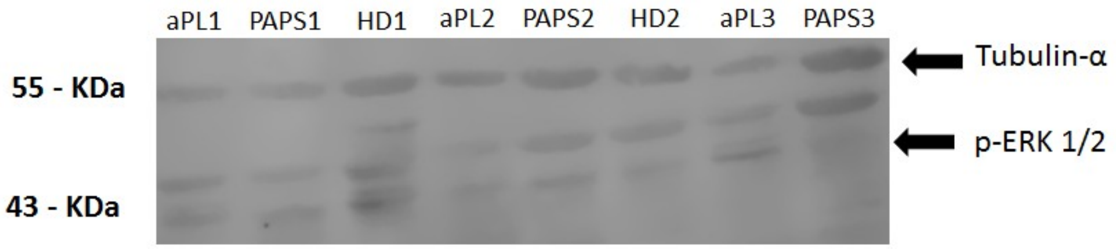

Supplementary Figure S8. In vitro gene expression of human primary monocytes. qPCR-RT was performed to evaluate the gene expression of several biological activation pathways in isolated human monocytes after incubation with sEVs from PAPS, aPL and healthy donors. No significant differences were observed in gene expression of p38 MAPK (*MAPK14*, *MAPK11),* MEK/ERK1 (*MAPK1, MAPK3, MA2PK1*), TLR-mediated signalling pathways (*TLR2/4, TRAF6*) and other relevant inflammatory cytokines (*TNFA, IFNA1*). One-way ANOVA was performed to analyse difference between three groups. sEVs: small extracellular vesicles; PAPS: primary antiphospholipid syndrome; aPL: positive antiphospholipid antibodies without thrombotic or obstetric complications, and HD: healthy donors.

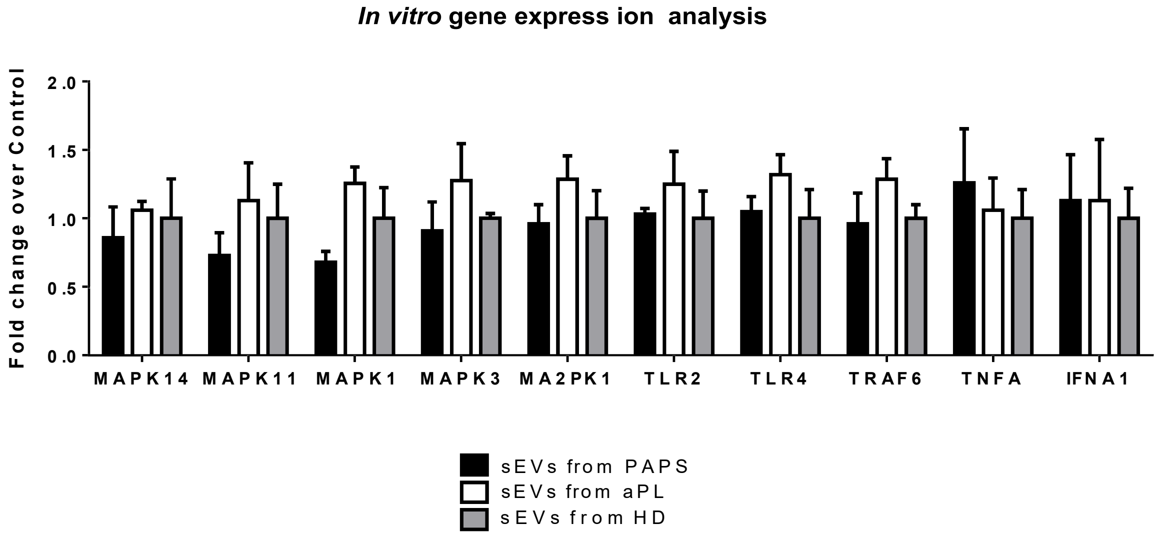

Supplementary Figure S9. miRNA expression analysis on HUVECS after transfection. Expression levels of miR-483-3p were evaluated by qPCR-RT after transfection with miR-483-3p mimic or control miR-mimic. Upregulation of miR-483-3p was observed in transfected miR-483-3p mimics HUVECs (Human Umbilical Vein Endothelial Cells). Relative expression was calculated using $2^{\Delta\text{-Ct}}$ and U6 snRNA were used as endogenous control. ***p<0.0001, t-student.

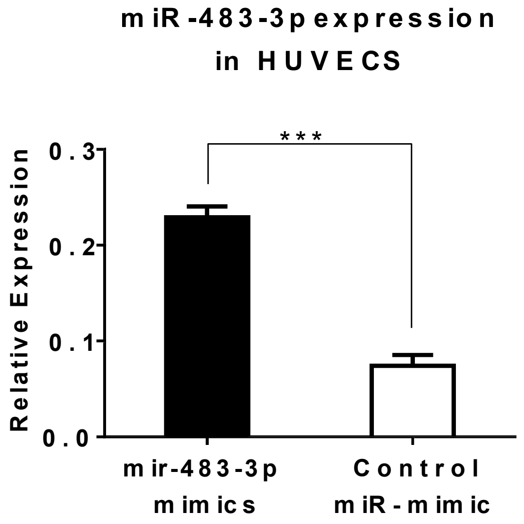

Supplementary Figure S10. Gene expression of IGF1, BBC3 and CDK4 in HUVECS. Cells were incubated with sEVs or transfected with miR-483-3p mimic or miR-mimic control. After 24 hours, gene expression of plausible target genes was evaluated. No significant differences were observed. Fold change was calculated over each control (for sEVs incubation: over sEVs from HD; for transfected cells: over control miR-mimic condition). sEVs: small extracellular vesicles; PAPS: with primary antiphospholipid syndrome; aPL: positive antiphospholipid antibodies without any thrombotic or obstetric complications and HD: healthy donors. One-way ANOVA was performed to analyse difference between groups.

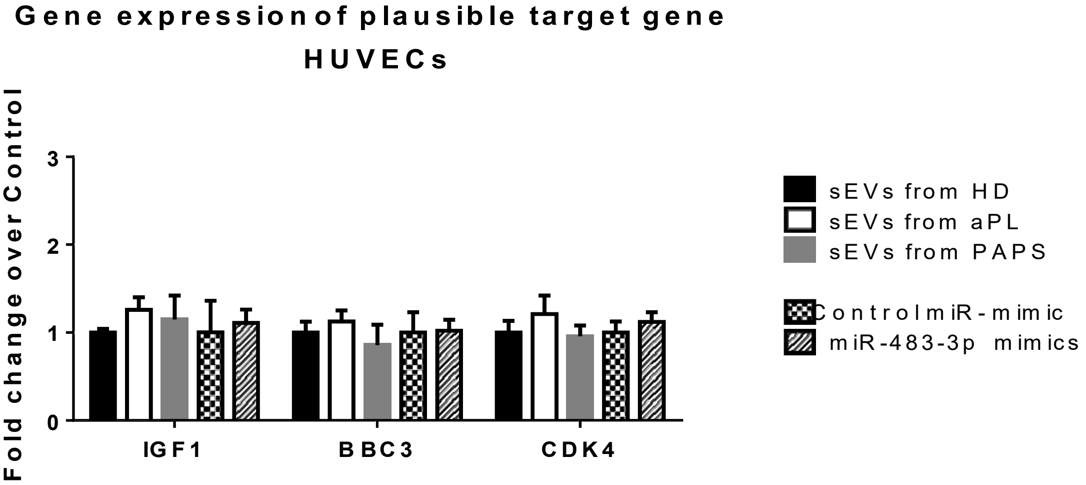

Supplementary Figure S11. In vitro gene expression in miR-483-3p mimic transfected monocytes. Overexpression of miR-483-3p in monocytes did not induce differential expression of tissue factor, NFKB1, IL12, MCP 1 and CD80. Relative expression was calculated using $2^{\Delta\text{-}Ct}$ and GADPH were used as endogenous control. No significant difference. t-student. F3: coagulation factor III or tissue factor; NFKB1: Nuclear factor kappa B subunit 1; IL12: interleukin-12; MCP1: Monocyte Chemoattractant Protein-1; CD80: T-lymphocyte activation antigen CD80.

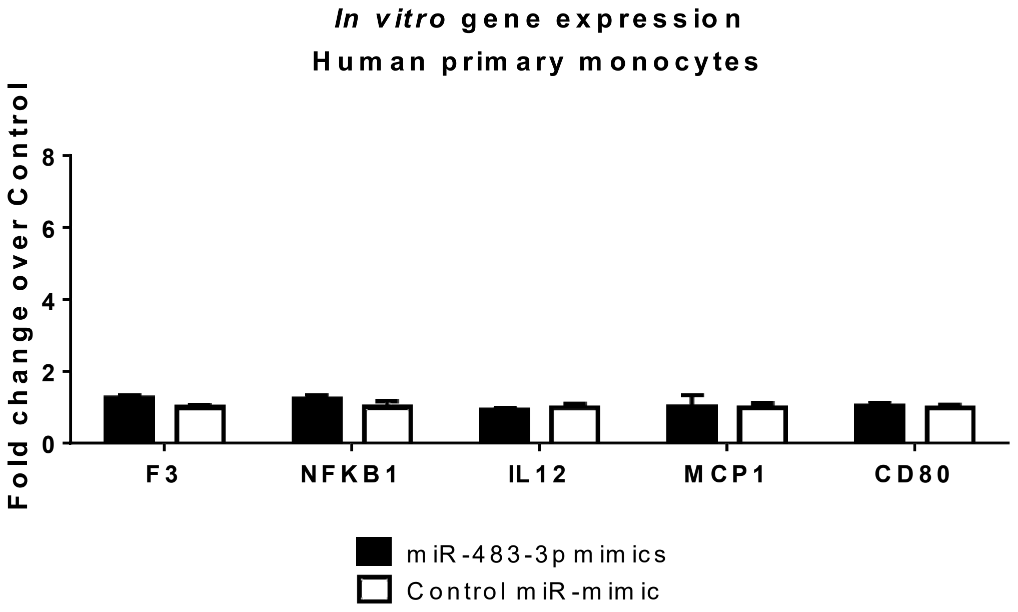

4. Supplementary Tables

Table S1. Main characteristics of patients with PAPS, aPL and HD included in the miRNA-seq screening study.

Characteristics	PAPS (N=8)	aPL (N=8)	HD (N=8)	P value ‡
**Female sex, *n* (%)**	5 (62.5)	6 (62.5)	6 (62.5)	0.817
**Median age (IQR), *y***	46 (39-58)	51 (47-55)	48 (42-55)	0.347
**Median duration of APS (IQR), *y***	8.5 (4-15)	10.0 (8-12)	na	0.780
**PAPS clinical criteria for initial anticoagulation, *n* (%)**				
Venous thrombosis	3 (37.5)	0 (0)	na	0.055
Arterial thrombosis	8 (100)	0 (0)	na	<0.001
Both arterial and venous	2 (25.0)	0 (0)	na	0.131
**Obstetrical medical history, *n* (%)**	3 (37.5)	0 (0)	na	0.055
**Other non-thrombotic manifestations, n (%)**				
Valvular heart Disease*	3 (37.5)	0 (0)	na	0.108
Renal thrombotic microangiopathy	2 (25.0)	0 (0)	na	0.131
Livedo reticularis	3 (37.5)	0 (0)	na	0.108
Migraine	3 (37.5)	0 (0)	na	0.108
Thrombocytopenia	3 (37.5)	2 (25.0)	na	0.589
**Laboratory profile at inclusion, *n* (%)**				
Lupus anticoagulant	8 (100)	8 (100)	na	1.000
Lupus anticoagulant alone	0 (0)	0 (0)	na	1.000
IgG/IgM antibodies				
aCL	8 (100)	8 (100)	na	1.000
Anti-β_2GPI	8 (100)	8 (100)	na	1.000
Anti-aPS/PT	8 (100)	8 (100)	na	1.000
**aCL/β_2GPI antibodies, mean (SD)**				
IgG aCL, *GPL units*	218.1 (180.5)	108.9 (73.1)	na	0.136
IgM aCL, *MPL units*	18.3 (22.9)	17.1 (8.3)	na	0.896
IgG anti-β_2GPI, *GPL units*	237.1 (273.1)	155.18 (81.5)	na	0.429
IgM anti-β_2GPI, *MPL units*	44.1 (95.9)	25.3 (19.9)	na	0.595
**Lupus anticoagulant and IgG aCL and IgG anti-β_2GPI antibodies, *n* (%)**	8 (100%)	8 (100%)	na	1.000
**GAPSS risk for thrombosis†**				
Mean score (SD)	14.2 (3.9)	13.1 (2.7)	na	0.517
Score, *n* (%)				
<10	2 (25.0)	1 (12.5)	na	1.000
10 to <15	2 (25.0)	4 (50.0)	na	0.608
≥15	4 (50.0)	3 (37.5)	na	1.000
**Aspirin use, *n* (%)**	4 (50.0)	6 (75.0)	na	0.608
**Vitamin K antagonist therapy, *n* (%)**	8 (100)	0 (0)	na	<0.001
**Coexisting cardiovascular risk factors, *n* (%)**				
Smoking	4 (50.0)	2 (25.0)	3 (37.5)	0.5866
Dyslipedemia	5 (62.5)	3 (37.5)	2 (25.0)	0.619
Diabetes mellitus	1 (12.5)	0 (0)	0 (0)	1.000
Hypertension	5 (62.5)	4 (50.0)	2 (25.0)	1.000

PAPS=Primary antiphospholipid syndrome. aPL: Patients with persistent antiphospholipid antibodies without thrombotic or obstetric complication; VKA= Vitamin K antagonist; aPS/PT=anti-phosphatidylserine/prothrombin antibodies; na= not applicable; n = Number; aCL = anticardiolipin; IgG= Immunoglobulin G; IgM= Immunoglobulin M; β_2GPI= Beta-2-glycopotein I; SD= Standard deviation; GAPSS = Global Anti-Phospholipid Syndrome Score; GPL = IgG phospholipid; IQR = interquartile range; MPL = IgM phospholipid. Disease duration: time from the diagnosis of the condition (PAPS or aPL) to the study sample. * Cardiac valvular disease was defined by an echocardiographic detection of lesions and/or regurgitation and/or stenosis of mitral and/or aortic valve according to the actual definition of APS-associated cardiac valve disease [3]. †GAPSS [29]. This is a categorical score derived from the combination of independent risk for thrombosis and pregnancy loss (PL), considering the aPL profile, conventional cardiovascular risk factors and the autoimmune antibody profile that is was developed and validated in a cohort of SLE patients. The GAPSS score ranges from 1 to 20, with higher scores indicating an increased risk. ‡ *P* value was calculated between groups, fisher exact test for categorial variables and 1-way ANOVA for continuous variables.

Table S2. The top five differentially expressed (DEGs) miRNAs founded in the microarray analysis of miRNAs-sEVs from PAPS, aPL and healty donor (N=8 for each group, p value and p adj value <0.05, logFC > |1.5|).

Comparison	miRNAs DEGs	Log FC	p value	p adj value
aPL vs HD	**hsa-miR-99a-5p**	5.72	<0.001	0.001
	**hsa-let-7a-3p**	4.76	<0.001	0.006
	**hsa-miR-3173-5p**	3.97	0.001	0.077
	**hsa-miR-532-3p**	3.03	0.001	0.077
	**hsa-miR-483-3p**	3.48	0.002	0.077
	hsa-miR-483-5p	-2.78	0.002	0.079
	hsa-miR-26a-5p	-1.92	<0.001	0.013
	hsa-miR-122-5p	1.60	0.003	0.079
	hsa-miR-122b-3p	1.60	0.003	0.079
PAPS vs HD	**hsa-miR-326**	-4.89	<0.001	0.008
	**hsa-let-7a-3p**	4.82	<0.001	0.008
	**hsa-miR-6741-5p**	-4.81	<0.001	0.008
	**hsa-miR-483-3p**	4.44	<0.001	0.010
	**hsa-miR-203a-3p**	4.40	0.001	0.041
	hsa-miR-203b-5p	4.40	0.001	0.041
	hsa-miR-320b	1.97	<0.001	0.008
	hsa-miR-191-5p	-1.95	<0.001	0.008
	hsa-miR-744-5p	-1.66	0.001	0.048
	hsa-miR-143-3p	-1.61	0.001	0.049
PAPS vs aPL	**hsa-miR-326**	-4.39	<0.001	0.126
	**hsa-miR-4433a-3p**	-3.45	0.001	0.126
	**hsa-miR-4433b-5p**	-3.45	0.001	0.126
	**hsa-miR-1-3p**	-3.34	0.001	0.126

Table S3. Target identification for miR-483-3p were obtained for three major miRNA-target datasets (miRecords, miRTarBase, and miRWalk) and using published manuscripts in Pubmed. We identified potential targets by described biological function (marked in bold).

Gene	Reference	Biological Function
SMAD4	Hao, J et al. MicroRNA 483-3p suppresses the expression of DPC4/Smad4 in pancreatic cancer. FEBS Lett. 2011; 585: 207-13.	Serves both as a transcription factor and as a tumor suppressor. Transcription factors help control the activity of particular genes, and tumor suppressors keep cells from growing and dividing too fast or in an uncontrolled way.
**BBC3**	Veronese, A et al. Mutated beta-catenin evades a microRNA-dependent regulatory loop. Proc Natl Acad Sci U S A. 2011; 108: 4840-5.	Essential mediator of p53/TP53-dependent and p53/TP53-independent apoptosis
**IGF1**	Ni, F et al. IGF-1 promotes the development and cytotoxic activity of human NK cells. Nat Commun. 2013; 4:1479.	Binds to the alpha subunit of IGF1R, leading to the activation of the intrinsic tyrosine kinase activity which autophosphorylates tyrosine residues in the beta subunit thus initiatiating a cascade of down-stream signaling events leading to activation of the PI3K-AKT/PKB and the Ras-MAPK pathways.
**PTEN**	Che, G et al. MicroRNA-483-3p promotes proliferation, migration, and invasion and induces chemoresistance of Wilms' tumor cells. Pediatr Dev Pathol. 2020; 23: 144-151.	Antagonizes the PI3K-AKT/PKB signaling pathway by dephosphorylating phosphoinositides and thereby modulating cell cycle progression and cell survival
**CDK4**	Bertero, T et al. CDC25A targeting by miR-483-3p decreases CCND-CDK4/6 assembly and contributes to cell cycle arrest. Cell Death Differ. 2013; 20:800-11.	Involved in cell cycle regulation (G1 to S transition),complexing with cyclin D (D1,D2,D3),amplified in malignant glioblastoma and anaplastic astrocytoma,and in diffuse large B cell lymphoma ; amplified in osteosarcoma with OS9,SAS and PRIM1 and in sporadic breast carcinomas,predisposing to familial melanoma by inducing abnormal repair of ultraviolet radiation. Loss of CDK4 expression causes insulin deficient diabetes,and CDK4 activation results in B islet cell hyperplasia in mice
APLNR	Zhang, C et al. Has_circ_0123190 acts as a competitive endogenous RNA to regulate APLNR expression by sponging hsa-miR-483-3p in lupus nephritis. Arthritis Res Ther. 2021; 23:24.	Receptor for apelin receptor early endogenous ligand (APELA) and apelin (APLN) hormones coupled to G proteins that inhibit adenylate cyclase activity.
SOX3	Yuan, L et al. LINC00662 promotes proliferation and invasion and inhibits apoptosis of glioma cells through miR-483-3p/SOX3 axis. Appl Biochem Biotechnol. 2022; 194: 2857-2871.	May function as a switch in neuronal development. Keeps neural cells undifferentiated by counteracting the activity of proneural proteins and suppresses neuronal differentiation. Required also within the pharyngeal epithelia for craniofacial morphogenesis. Controls a genetic switch in male development. Is necessary for initiating male sex determination by directing the development of supporting cell precursors (pre-Sertoli cells) as Sertoli rather than granulosa cells.
GDF3	Ferland-McCollough, D et al. Programming of adipose tissue miR-483-3p and GDF-3 expression by maternal diet in type 2 diabetes. Cell Death Differ. 2012; 19: 1003-12.	Growth factor involved in early embryonic development and adipose-tissue homeostasis. During embryogenesis controls formation of anterior visceral endoderm and mesoderm and the establishment of anterior-posterior identity through a receptor complex comprising the receptor ACVR1B and the coreceptor TDGF1/Cripto. Regulates adipose-tissue homeostasis and energy balance under nutrient overload in

		part by signaling through the receptor complex based on ACVR1C and TDGF1/Cripto
CDC25A	Bertero, T et al. CDC25A targeting by miR-483-3p decreases CCND-CDK4/6 assembly and contributes to cell cycle arrest. Cell Death Differ. 2013; 20:800-11.	Tyrosine protein phosphatase which functions as a dosage-dependent inducer of mitotic progression. Directly dephosphorylates CDK1 and stimulates its kinase activity. Also dephosphorylates CDK2 in complex with cyclin E, in vitro.
API5	Bertero, T et al. Tumor suppressor function of miR-483-3p on squamous cells carcinomas due to its pro-apoptotic properties. Cell Cycle. 2013; 12:2183-93.	Antiapoptotic factor that may have a role in protein assembly. Negatively regulates ACIN1. By binding to ACIN1, it suppresses ACIN1 cleavage from CASP3 and ACIN1-mediated DNA fragmentation. Also known to efficiently suppress E2F1-induced apoptosis. Its depletion enhances the cytotoxic action of the chemotherapeutic drugs.

Table S4. Target identification for miR-326 were obtained for three major miRNA-target datasets (miRecords, miRTarBase, and miRWalk) and using published manuscripts in Pubmed. We identified potential targets by described biological function (marked in bold).

Gene	Reference	Biological Function
GLI1	Ferreti E, et al. Concerted microRNA control of Hedgehog signalling in cerebellar neuronal progenitor and tumor cells. EMBO J. 2008; 27:2616-27.	A transcriptional regulator involved in the development of different types of cancer. GLI1 transcriptional activity is regulated within the Hedgehog pathway (canonical activity), but can also be controlled independently (non-canonical activity) in the context of other signaling pathways.
SMO	Jiang Z, et al. miR-326 is downstream of Sonic hedgehog signalling and regulates the expression of Gli2 and smoothened. Am J Respir Cell Mol Biol 2014; 51:273-83.	Mediates signal transduction in the hedgehog pathway, which is implicated in normal development and carcinogenesis.
**NOTCH1**	Kefas B, et al. The neuronal microRNA miR-326 acts in a feedback loop with notch and has therapeutic potential against brain tumors. J Neurosci. 2009; 29:15161-8.	Role in cell growth and division (proliferation), maturation (differentiation), and self-destruction (apoptosis).
PKM	Wu H, et al. Kaempferol can reverse the 5-fu resistance of colorectal cancer cells by inhibiting PKM2-mediated glycolysis. Int J Mol Sci. 2022; 23:3544.	Encoded protein is a pyruvate kinase that catalyzes the transfer of a phosphoryl group from phosphoenolpyruvate to ADP, generating ATP and pyruvate.
HOTAIR	Ke J, et al. Knockdown of long non-conding RNA HOTAIR inhibits malignant biological behaviors of human glioma cells via modulation of miR-326. Oncotarget. 2015; 6:21934-49.	HOTAIR has been shown to function as a key regulator of chromatin states and dynamics by binding to the specific chromatin modification complex polycomb repressive complex 2 (PRC2), thereby recruiting and affecting PRC2 occupancy on genes genome-wide
**FGF1**	Ren, H et al. MiR-326 antagomir delays the progression of age-related cataract by upregulating FGF1-mediated expression of betaB2-crystallin. Biochem Biophys Res Commun. 2018; 505:505-510.	Protect the cell against stress conditions by providing an additional signal for cell survival, independently of receptor-activated signaling cascades
FSCN1	Li, Y et al. Down-regulation of miR-326 is associated with poor prognosis and promotes growth and metastasis by targeting FSCN1 in gastric cancer. Growth Factors. 2015; 33: 267-74.	Organize F-actin into parallel bundles, and are required for the formation of actin-based cellular protrusions. The encoded protein plays a critical role in cell migration, motility, adhesion and cellular interactions
PRDM16	Wang, J et al. Development and validation of a novel circular RNA as an independent prognostic factor in actue myeloid leukemia. BMC Med. 2021; 19:28.	Controls the cell fate between muscle and brown fat cells.
**ZEB1**	Liu, M et al. MiR-326 mediates malignant biological behaviors of lung adenocarcinoma by targeting ZEB1. Sci Prog. 2021; 104: 368504211009379.	ZEB1 is suppressing the expression of its target genes, such as epithelial markers (E-cadherin), and correspondingly increasing the mesenchymal levels of vimentin and N-cadherin
HNRNPA2B1	Luo, J et al. LncRNA PCAT6 facilitates cell proliferation and	Important roles in initiating IFN-alpha/beta production and enhancing stimulator of interferon genes (STING)-

	invasion via regulating the miR-326/hnRNPA2B1 axis in liver cancer. Oncol Lett. 2021; 21: 471.	dependent cytoplasmic antiviral signaling
**ITGA5**	Wang, P et al. SNHG3 silencing suppresses the malignant development of triple-negative breast cancer cells by regulating miRNA-326/integrin α5 axis and inactivating Vav2/Rac1 signaling pathway. Eur Rev Med Pharmacol Sci. 2020; 24: 5481-5492.	Integrins are known to participate in cell-surface mediated signalling
ETV4	Qi, Y et al. Circular RNA hsa_circ_0001666 sponges miR-330-5p, miR-193a-5p and miR-326, and promotes papillary thryroid carcinoma progression via upregulation of ETV4. Oncol Rep. 2021; 45: 50.	Control the development of various organs and are involved in the progression of many cancers, including breast cancer
HIPK3	Zhuang, L et al. CircHIPK3 alleviates high glucose toxicity to human renal tubular epithelial HK-2 cells through regulation of miR-326/miR-487a-3p/SIRT1. Diabetes Metab Syndr Obes. 2021; 14: 729-740.	Serine/threonine-protein kinase involved in transcription regulation, apoptosis and steroidogenic gene expression
TFF1	Pan, G et al. Circular RNA has_circ_0061828 (circ-TFF1) contributes to breast cancer progression through targeting miR-326/TFF1 signalling. Cell Prolif. 2020; 53: e12720.	Protect the mucosa from insults, stabilize the mucus layer, and affect healing of the epithelium.

Table S5. Fluorescent labelled antibodies used for flow cytometry analysis

Antibody	Fluorescent	Supplier	Code
Anti-Human CD41a	APC	BD Biosciences	561852
Anti-Human CD31	PE	BD Biosciences	566177
Anti-Human CD16	FITC	BD Biosciences	347523
Anti-Human CD62P	PE	BD Biosciences	348107

Table S6. Primer IDs used in Taqman RT-qPCR from Applied Biosystems.

miRNAs	Assay ID (TaqMan)
**miR-93-5p (control)**	478210_mir
**miR-483-3p**	478122_mir
**miR-326**	478027_mir

Gene	Assay ID (TaqMan)
**GADPH**	Hs02786624_g1
**mTOR**	Hs00234508_m1
**PIK3CA**	Hs00907957_m1
**AKT1**	Hs00178289_m1
**EDN1**	Hs00174961_m1
**ICAM1**	Hs00164932_m1
**VCAM1**	Hs01003372_m1
**NFKB1**	Hs00765730_m1
**F3 (tissue factor)**	Hs01076029_m1
**TGFB1**	Hs00998133_m1
**TGFBR1**	Hs00610320_m1
**SMAD2**	Hs00998187_m1
**SMAD3**	Hs00969210_m1
**MAP2K1**	Hs05512159_s1
**MAPK3**	Hs00385075_m1
**MAPK1**	Hs01046830_m1
**MAPK8**	Hs01548508_m1
**TRAF6**	Hs00939742_g1
**TLR2**	Hs02621280_s1
**TLR4**	Hs00152939_m1
**SELE**	Hs00174057_m1
**IL1A**	Hs00174092_m1
**TNFA**	Hs00174128_m1
**IL6**	Hs00174131_m1
**IL8**	Hs00174103_m1
**IFNA1**	Hs03044218_g1
**IFNB**	Hs01077958_s1
**MAPK14**	Hs01051152_m1
**MAPK11**	Hs00177101_m1
**CCL2 (MCP1)**	Hs00234140_m1
**IL12A**	Hs01073447_m1
**CD80**	Hs01045161_m1
**PTEN**	Hs02621230_s1
**NOTCH1**	Hs01062014_m1
**IGF1**	Hs01547656_m1
**BBC3**	Hs00248075_m1
**CDK4**	Hs00364847_m1

Table S7. Primary and secondary antibodies used for immunofluorescence

Primary Antibody	Supplier	Code
**Anti-VCAM1**	Abcam	ab134047
**Anti-ICAM1**	Abcam	Ab171123
**Anti-p65-NFKB**	Genetex	GTX102090
**Secondary Antibody**	**Supplier**	**Code**
**Alexa-488-conjugated anti-rabbit IgG**	Abcam	ab150077
**Alexa-647-conjugated anti-mouse IgG**	Abcam	ab150107
